# *Trans*-lesional fractional flow reserve gradient as derived from coronary CT improves patient management: ADVANCE registry^☆^

**DOI:** 10.1016/j.jcct.2021.08.003

**Published:** 2021-09-02

**Authors:** Hidenobu Takagi, Jonathon A. Leipsic, Noah McNamara, Isabella Martin, Timothy A. Fairbairn, Takashi Akasaka, Bjarne L. Nørgaard, Daniel S. Berman, Kavitha Chinnaiyan, Lynne M. Hurwitz-Koweek, Gianluca Pontone, Tomohiro Kawasaki, Niels Peter Rønnow Sand, Jesper M. Jensen, Tetsuya Amano, Michael Poon, Kristian A. Øvrehus, Jeroen Sonck, Mark G. Rabbat, Sarah Mullen, Bernard De Bruyne, Campbell Rogers, Hitoshi Matsuo, Jeroen J. Bax, Pamela S. Douglas, Manesh R. Patel, Koen Nieman, Abdul Rahman Ihdayhid

**Affiliations:** a Department of Radiology, St. Paul’s Hospital and University of British Columbia, Vancouver, British Columbia, Canada; b Department of Radiology, Iwate Medical University Hospital, Iwate, Japan; c Department of Diagnostic Radiology, Tohoku University Hospital, Miyagi, Japan; d Department of Cardiology, Liverpool Heart and Chest Hospital, University of Liverpool, Liverpool, UK; e Department of Cardiovascular Medicine, Wakayama Medical University, Wakayama, Japan; f Department of Cardiology, Aarhus University Hospital, Aarhus, Denmark; g Division of Nuclear Imaging, Department of Imaging, Cedars-Sinai Heart Institute, Los Angeles, CA, USA; h Division of Cardiology, Beaumont Academic Heart and Vascular Group, Royal Oak, MI, USA; i Division of Cardiology, Department of Medicine, Duke University Medical Center, Duke Clinical Research Institute, Duke University School of Medicine, Durham, NC, USA; j Centro Cardiologico Monzino, IRCCS, Milan, Italy; k Cardiovascular Center, Shin Koga Hospital, Fukuoka, Japan; l Cardiac Research Unit, Institute of Regional Health Research, University Hospital of Southern DK, Esbjerg and University of Southern DK, Denmark; m Department of Cardiology, Aichi Medical University, Aichi, Japan; n Department of Noninvasive Cardiac Imaging, Northwell Health, New York, NY, USA; o Department of Cardiology, Odense University Hospital, Denmark; p Cardiovascular Center Aalst, OLV Clinic, Aalst, Belgium; q Department of Advanced Biomedical Sciences, University of Naples Federico II, Naples, Italy; r Division of Cardiology, Loyola University Chicago, Chicago, IL, USA; s HeartFlow Inc., Redwood City, CA, USA; t Department of Cardiology, University Hospital of Lausanne, Lausanne, CH, USA; u Department of Cardiovascular Medicine, Gifu Heart Center, Gifu, Japan; v Department of Cardiology, Leiden University Medical Center, Leiden, the Netherlands; w Department of Cardiovascular Medicine and Radiology, Stanford University, Stanford, CA, USA; x Department of Cardiology, Fiona Stanley Hospital, Harry Perkins Institute of Medical Research, University of Western Australia, Perth, Australia

**Keywords:** Coronary artery disease (CAD), Fractional flow reserve (FFR), Coronary computed tomography angiography (CCTA), Fractional flow reserve derived from coronary computed tomography (FFR_CT_)

## Abstract

**Background:**

The role of change in fractional flow reserve derived from CT (FFR_CT_) across coronary stenoses (ΔFFR_CT_) in guiding downstream testing in patients with stable coronary artery disease (CAD) is unknown.

**Objectives:**

To investigate the incremental value of ΔFFR_CT_ in predicting early revascularization and improving efficiency of catheter laboratory utilization.

**Materials:**

Patients with CAD on coronary CT angiography (CCTA) were enrolled in an international multicenter registry. Stenosis severity was assessed as per CAD-Reporting and Data System (CAD-RADS), and lesion-specific FFR_CT_ was measured 2 cm distal to stenosis. ΔFFR_CT_ was manually measured as the difference of FFR_CT_ across visible stenosis.

**Results:**

Of 4730 patients (66 ± 10 years; 34% female), 42.7% underwent ICA and 24.7% underwent early revascularization. ΔFFR_CT_ remained an independent predictor for early revascularization (odds ratio per 0.05 increase [95% confidence interval], 1.31 [1.26–1.35]; *p* < 0.001) after adjusting for risk factors, stenosis features, and lesion-specific FFR_CT_. Among the 3 models (*model 1*: risk factors + stenosis type and location + CAD-RADS; *model 2*: model 1 + FFR_CT_; *model 3*: model 2 + ΔFFR_CT_), model 3 improved discrimination compared to model 2 (area under the curve, 0.87 [0.86–0.88] vs 0.85 [0.84–0.86]; *p* < 0.001), with the greatest incremental value for FFR_CT_ 0.71–0.80. ΔFFR_CT_ of 0.13 was the optimal cut-off as determined by the Youden index. In patients with CAD-RADS ≥3 and lesion-specific FFR_CT_ ≤0.8, a diagnostic strategy incorporating ΔFFR_CT_ >0.13, would potentially reduce ICA by 32.2% (1638–1110, *p* < 0.001) and improve the revascularization to ICA ratio from 65.2% to 73.1%.

**Conclusions:**

ΔFFR_CT_ improves the discrimination of patients who underwent early revascularization compared to a standard diagnostic strategy of CCTA with FFR_CT_, particularly for those with FFR_CT_ 0.71–0.80. ΔFFR_CT_ has the potential to aid decision-making for ICA referral and improve efficiency of catheter laboratory utilization.

## 1. Introduction

Physiological assessment with fractional flow reserve (FFR) guides the revascularization in patients with stable coronary artery disease (CAD).^1,2^ The application of computational fluid dynamics to a standard coronary computed tomography angiography (CCTA) enables non-invasive FFR measurement (FFR_CT_) without additional imaging, medications, radiation exposure, or hospital visits.^3^ Numerous studies have demonstrated the diagnostic performance,^4,5^ prognostic value,^6,7^ and clinical utility of FFR_CT_ in real-world practice.^7–9^ FFR_CT_ is derived along the epicardial coronary tree. This allows for a flexible and lesion-specific approach that goes beyond the standard assessment which focuses on whether vessel specific FFR_CT_ falls below a specific cut-off. The change in FFR_CT_ values across a stenosis (ΔFFR_CT_) represents an estimate of lesion-specific pressure loss and have been shown to discriminate a more focal phenotype of physiology and identify high risk plaques.^10,11^

The ADVANCE (Assessing Diagnostic Value of Non-invasive FFR_CT_ in Coronary Care) registry (NCT02499679) is an international multicenter prospective registry that enrolled stable patients with CAD who were investigated with CCTA and FFR_CT_.^7,8^ More than half of patients who underwent ICA in gray-zone FFR_CT_ value did not receive subsequent revascularization in the ADVANCE registry. There could be space to utilize FFR_CT_ beyond the standard measurement of FFR_CT_ in terms of catheter laboratory utilization. In this analysis, we hypothesized that ΔFFR_CT_ would improve the identification of those who required early revascularization and investigated the incremental value of ΔFFR_CT_ at improving the efficiency of downstream invasive testing as assessed by the revascularization to invasive coronary angiography (ICA) ratio.

## 2. Materials and methods

### 2.1. Study design and population

The design and outcomes of the ADVANCE registry have been described previously.^7,8^ Patients being investigated for clinically suspected CAD with documented >30% stenosis on CCTA were prospectively enrolled at 38 sites in Europe, Japan, and North America from July 2015, to October 2017. Exclusion criteria were poor CCTA image quality, life expectancy <1-year, or an inability to comply with follow-up requirements. The decision to request an FFR_CT_ analysis was independently determined by the clinician reporting the CCTA. All patients provided written informed consent following institutional review board review and approval. In this secondary analysis, patients not referred for FFR_CT_ analysis or in whom FFR_CT_ was unanalyzable or unavailable were excluded (Supplemental Figure 1).

### 2.2. CCTA acquisition and interpretation

CCTA was performed as per local practice and international guidelines.^12,13^ The sites investigators graded coronary stenosis severity as normal, 0%–29%, 30%–49%, 50%–69%, 70%–90%, >90%, occluded (100%). For this sub-analysis, the per-patient anatomical severity was classified according to the Coronary Artery Disease – Reporting and Data System (CAD-RADS^™^) (Supplemental Table 1).^14^ This evaluation did not include high-risk plaque findings in this study.

### 2.3. FFR_CT_ analysis and measurements

The analysis was blindly performed at HeartFlow (Redwood, CA, United States). For all patients, 3-dimensional anatomic models of epicardial coronary arteries and aortic root were generated from CCTA images.

**Fig. 1.**
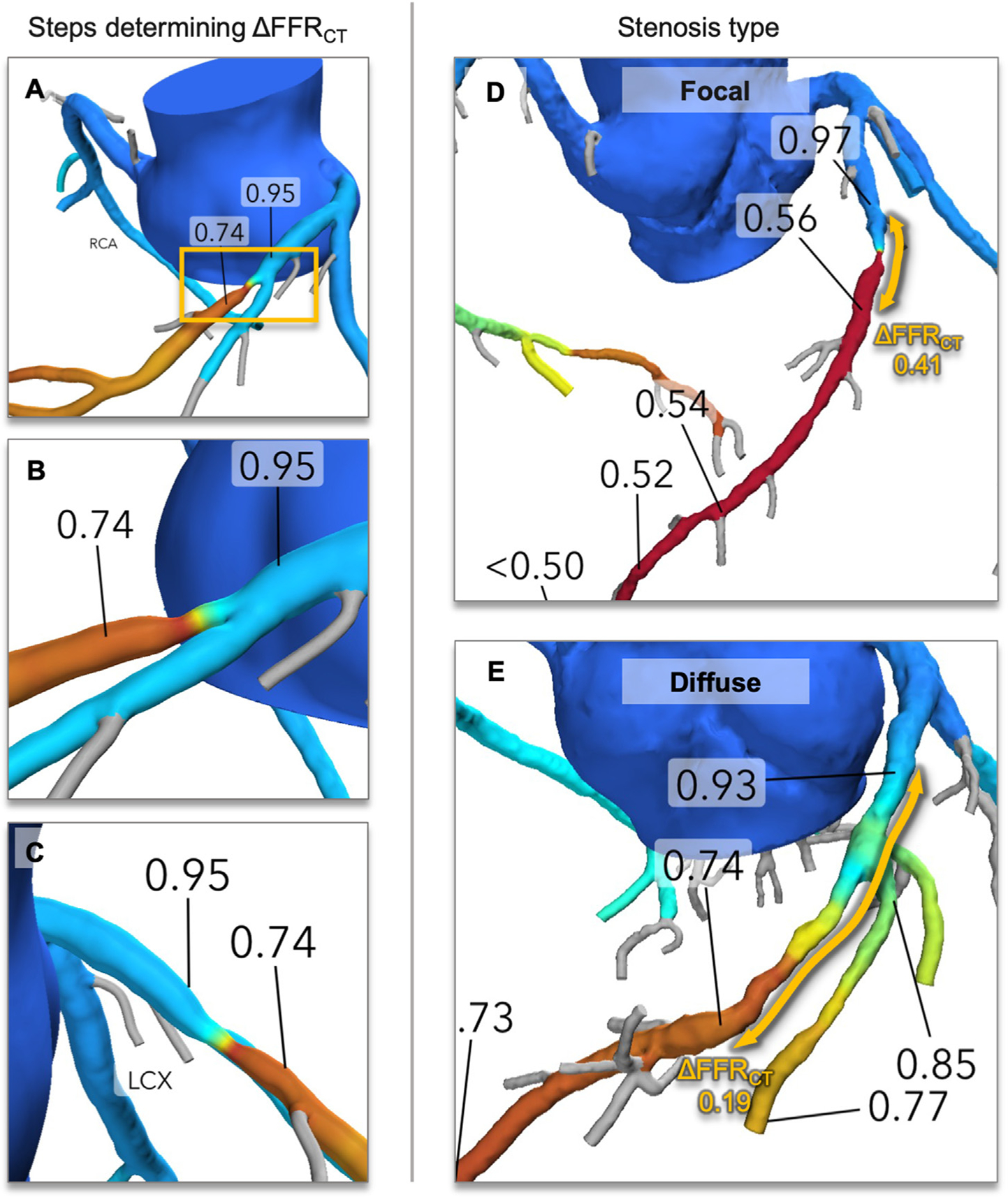
Methodology for determining ΔFFRCT and stenosis type. First the presence of and extent of stenosis is visually determined by analysing the 3-dimensional (3D) model in multiple projections. Proximal and distal reference points are then markedon the 3D model at regions immediately adjacent to the stenosis at regions which appear free of luminal stenosis (B and C). The ΔFFRCT was defined as the difference of FFRCT values between these two points.The stenosis type for each ΔFFRCT measurement was visually categorized as *focal* or *diffuse*, based on lesion length visually assessed on the 3D coronary model as follows (D and E).

In accordance with the expert consensus for interpretation of FFR_CT_,^15^ we obtained both lesion-specific FFR_CT_ and ΔFFR_CT_ for each coronary vessel using the patient-specific 3-dimensional FFR_CT_ model. A central core laboratory (Duke Clinical Research Institute, Durham, NC, United States) blinded to clinical information reviewed all FFR_CT_. Lesion-specific FFR_CT_ was measured at 2 cm distal to stenosis for each coronary artery.^15^ An FFR_CT_ of ≤0.8 was defined as a positive value. Additional analyses, blinded to clinical information, were performed in our core laboratory (St. Paul’s Hospital, Vancouver, BC, Canada), where we reviewed all FFR_CT_ models and measured ΔFFR_CT_. The ΔFFR_CT_ represents the change in FFR_CT_ across a stenosis and was measured as the difference in FFR_CT_ values proximal and distal to a stenosis. The proximal and distal reference points were both manually identified at the most adjacent points to visible stenosis on the 3-dimensional FFR_CT_ model (Fig. 1).^16^ The distance between the proximal and distal ΔFFR_CT_ reference points was visually assessed to characterize the stenosis type (Fig. 1D–F):

- Focal – length <1 coronary segment, assuming <39 mm
- Diffuse – length >1 segment, assuming ≥40 mm

In a case with diffuse stenosis, after placing proximal reference at the most adjacent to the visible stenosis, carefully looked along downstream coronary and placed the distal reference at the point with visually normal diameter being the most adjacent to stenosis. The reproducibility of ΔFFR_CT_ was excellent (Supplemental document). The lesion location was determined according to the Society of Cardiovascular Computed Tomography guidelines.^12^ Per-patient lesion-specific FFR_CT_ was recorded as the lowest lesion-specific FFR_CT_ in major epicardial coronary arteries, and ΔFFR_CT_ associated with the minimum lesion-specific FFR_CT_ was deemed per-patient ΔFFR_CT_.

### 2.4. Patient management and clinical end points

The site investigator and the institution’s heart team reviewed clinical data and interpreted all available diagnostic tests, including CCTA and FFR_CT_. The clinical management decisions including revascularization or medical therapy, entirely rested with the site physician and heart team.^17^ Early revascularization was defined as percutaneous coronary intervention (PCI) or coronary artery bypass grafting (CABG) performed within 90 days after enrollment.^8,17^ Patients who did not undergo early revascularization were deemed to have undergone medical therapy alone.

The primary endpoint of this study was the early revascularization. The secondary endpoints were the number of ICA and the ratio of early revascularization.

### 2.5. Statistical analysis

Descriptive statistics were presented as mean ± standard deviation for continuous variables and raw number (percentages) for categorical variables. Independent variables were compared using unpaired t or Fisher’s exact test as appropriate. Multivariable logistic regression analysis was conducted to assess the association between ΔFFR_CT_ and early revascularization. The multivariable adjustment was performed for clinical risk factors (age, sex, symptom status, hypertension, diabetes, hyperlipidemia, and current smoking), CAD-RADS, lesion-specific FFR_CT_, lesion location, and stenosis type, and the interaction between lesion-specific FFR_CT_ and ΔFFR_CT_. Heterogenicity of the relationship between ΔFFR_CT_ and early revascularization was assessed according to subgroups including symptom, CAD-RADS, lesion-specific FFR_CT_, stenosis location, and stenosis type. Three models were created to assess the incremental value of ΔFFR_CT_ to a standard CCTA with FFR_CT_ strategy: *model 1*, risk factors + CAD-RADS + stenosis type and location; *model 2, model 1* + lesion-specific FFR_CT_; and *model 3, model 2* + ΔFFR_CT_. The area under the curve (AUC) was compared using DeLong’s test.^18^ Heterogenicity of the incremental value was assessed according to CAD-RADS and lesion-specific FFR_CT_ severity. A 2-sided *p*-value of <0.05 was considered statistically significant in all tests. Computation was performed using JMP PRO version 14 (SAS Institute Inc., Cary, NC, USA) or R version 4.1 (R Foundation, Vienna, Austria).

### 2.6. Simulation of efficacy of ICA referral

We conducted an ICA referral simulation to assess the impact of ΔFFR_CT_ on the efficiency of catheter laboratory utilization. We randomly selected 2839 (60.0%) patients for determining the cut-off value of ΔFFR_CT_ according to the Youden index and validated the cut-off value with the remaining patients. This analysis allows for the greatest extent of confirmation possible without a separate cohort. Subsequently, we simulated referral for ICA according to three potential strategies: *Anatomical*, ICA referral for patients with CAD-RADS ≥3; *Lesion-specific FFR*_*CT*_, CAD-RADS ≥3 and lesion-specific FFR_CT_ ≤0.80; and *ΔFFR*_*CT*_, CAD-RADS ≥3, lesion-specific FFR_CT_ ≤0.80, and ΔFFR_CT_ > cut-off value. To account for other clinical factors related to a decision for early revascularization, we applied this simulation to patients who underwent ICA, meaning that more likely to undergoing ICA. For each of these strategies, the potential impact of ΔFFR_CT_ at reducing the number of ICA and improving the ratio of subsequent revascularization was assessed.

## 3. Results

### 3.1. Patient characteristics

Of the 5083 patients enrolled in the registry, FFR_CT_ analysis was requested in 4893 (96.2%). FFR_CT_ analysis was feasible in 4737 (93.2%) and accessible for this sub-analysis in 4730 (93.1%) (Supplemental Figure 1). A total of 2092 (42.7%) patients underwent ICA within 90 days, with 1168 (24.7%) patients requiring early revascularization (PCI: 1017 [87.1%]; CABG 151 [22.9%]). Patients who underwent revascularization were more likely to be male and to have typical angina, hypertension, diabetes mellites, hyperlipidemia, and active smoking (Table 1).

### 3.2. Relationship of CAD severity with actual treatment

**Fig. 2.**
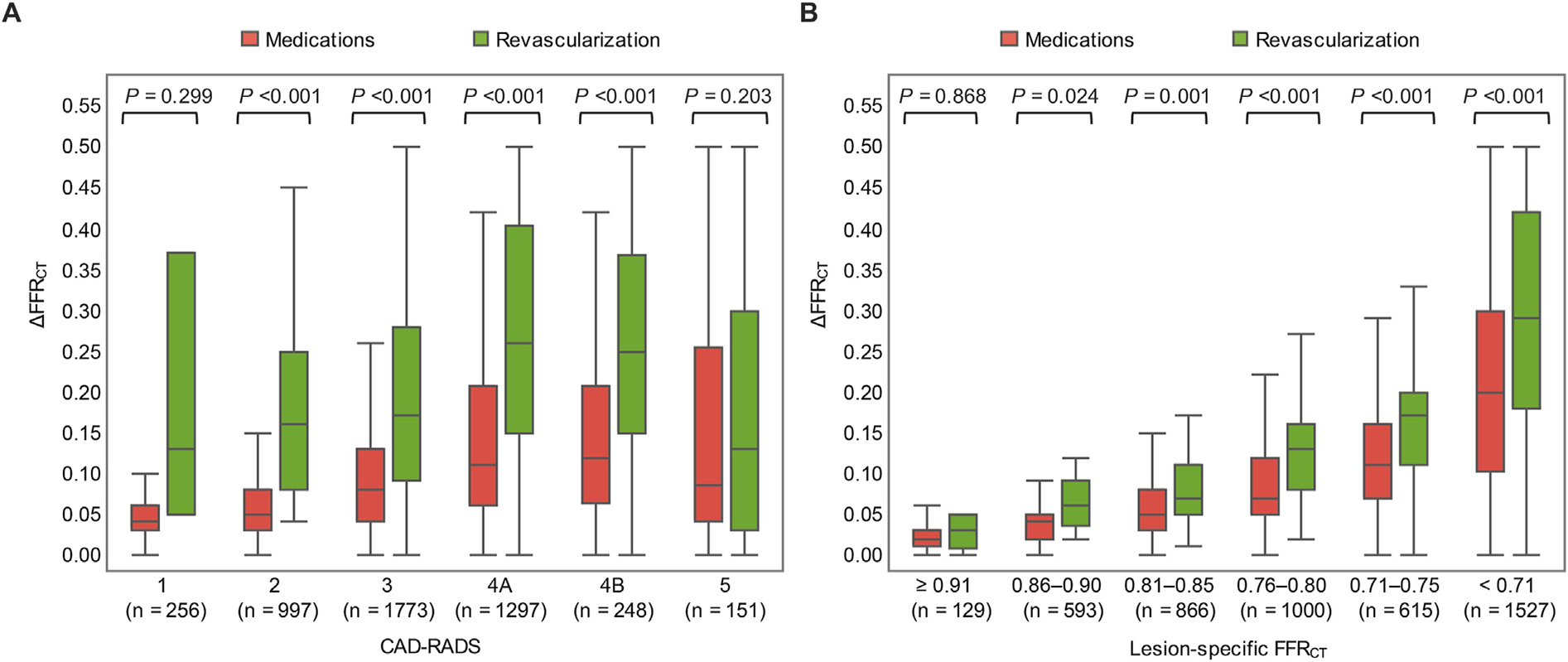
Relationship of ΔFFR_CT_ with CAD-RADS (A) and lesion-specific FFR_CT_ (B). ΔFFR_CT_ was compared between patients with vs. without early revascularization in CAD-RADS (A) and FFR_CT_ category (B).

Table 2 summarizes anatomical and physiological CAD characteristics. Patients with early revascularization showed higher CAD-RADS grading, as well lower lesion-specific FFR_CT_ and larger ΔFFR_CT_. A larger ΔFFR_CT_ was observed with increasing stenosis severity (Supplemental Figure 2A); further, a larger ΔFFR_CT_ was observed in patients with early revascularization across each anatomical severity (Fig. 2A). A larger ΔFFR_CT_ was associated with lower lesion-specific FFR_CT_ (Supplemental Figure 2B); furthermore, a larger ΔFFR_CT_ was observed in patients requiring early revascularization across each group stratified by 0.05 increments in lesion-specific FFR_CT_ (Fig. 2B).

**Fig. 3.**
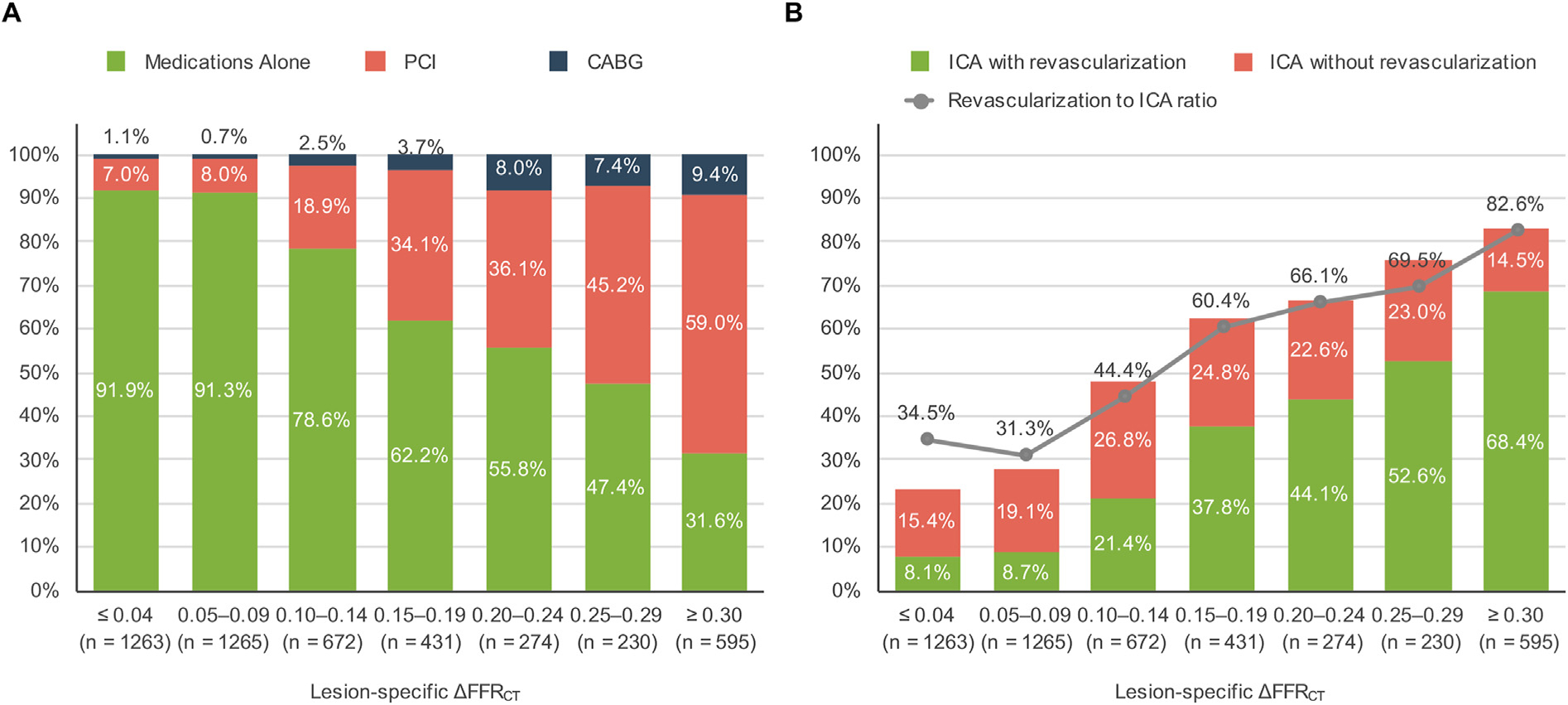
Relationship of ΔFFR_CT_ with actual treatment at 90 days (A) and ICA results (B). Panel A shows actual treatment, including medications alone, percutaneous coronary intervention (PCI), or coronary artery bypass grafting (CABG) stratified by 0.05 ΔFFR_CT_ increments. Panel B shows the ratio of ICA with or without revascularization and the ratio of revascularization to ICA stratified by 0.05 ΔFFR_CT_ increments.

Early revascularization was associated with a larger ΔFFR_CT_ as compared to patients treated medically (0.24 ± 0.15 vs. 0.10 ± 0.09; *p* < 0.001). With increasing ΔFFR_CT_, patients were more likely to undergo ICA and revascularization and were associated with an increase in the revascularization to ICA ratio (Fig. 3). The revascularization rate in patients with CAD-RADS 3 and ≥ 4 was 15.6% (276/1773) and 50.6% (858/1696), respectively. Patients with a lesion-specific FFR_CT_ of >0.80, 0.71–0.80, and ≤0.70 underwent revascularization at a rate of 4.4% (70/1588), 17.0% (275/1615), and 53.9% (823/1527), respectively.

### 3.3. ΔFFR_CT_ as an independent predictor for early revascularization

**Fig. 4.**
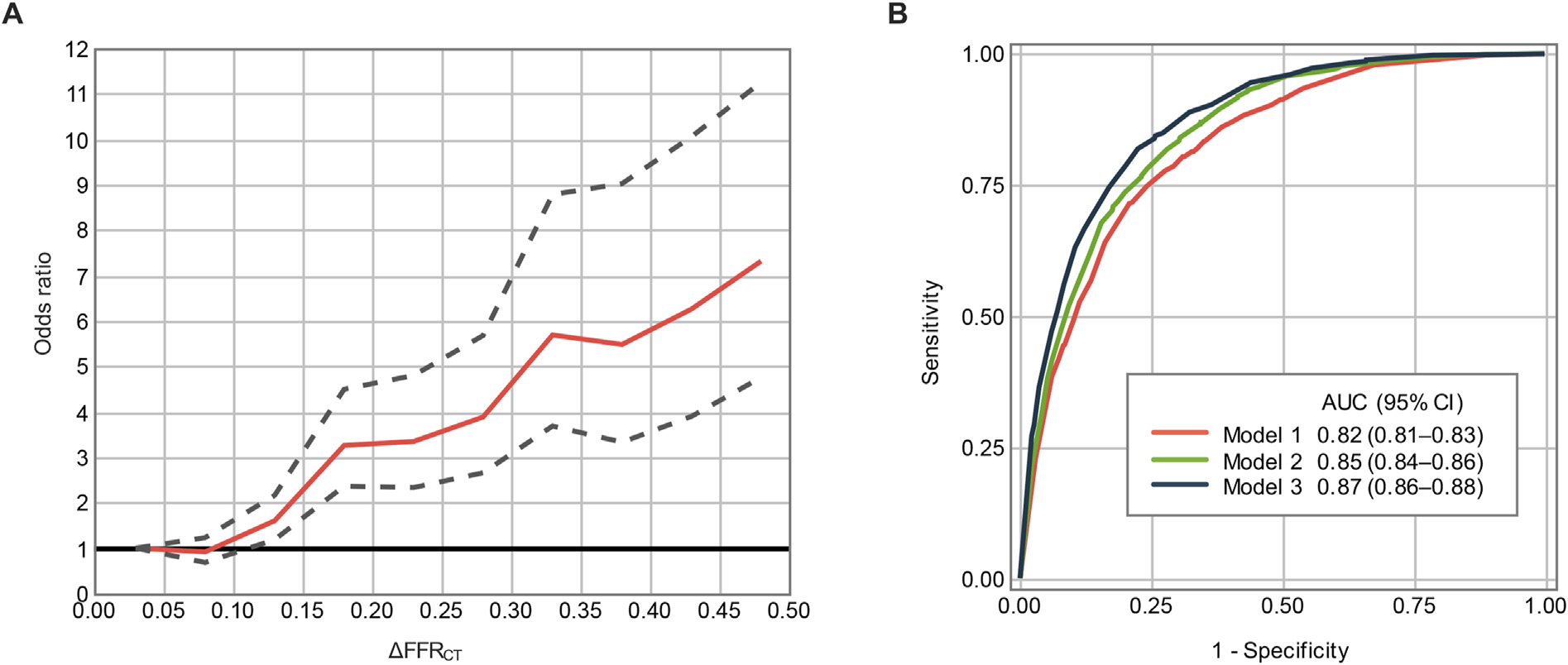
Multivariable logistic regression analysis for predicting early revascularization. Panel A shows odds ratio (solid line) with 95% confidence interval (dotted line) of ΔFFR_CT_ compared with ΔFFR_CT_ of 0.00–0.04 after adjusting risk factors, CAD-RADS, stenosis type and location, and FFR_CT_.Panel B shows receiver operating characteristic curves for three logistic models for early revascularization: *model 1* = risk factors, CAD-RADS, stenosis type and location; *model 2* = *model 1* + FFR_CT_; and *model 3* = *model 2* + ΔFFR_CT_. *Model 2* demonstrated higher AUC as compared to *model 1* (AUC difference with 95% CI, 0.02 [0.02–0.03], *p* < 0.001). Model 3 demonstrated the highest AUC and was superior to *model 1* (0.05 [0.04–0.05], *p* < 0.001) and *model 2* (0.02 [0.02–0.03], *p* < 0.001).AUC = area under the curve; CI = confidence interval.

ΔFFR_CT_ remained an independent predictor for early revascularization after adjusting for age, sex, hypertension, hyperlipidemia, diabetes mellites, angina status, CAD-RADS, stenosis type and location, and FFR_CT_ (Table 3). The adjusted odds ratio for early revascularization per 0.05-unit increase in ΔFFR_CT_ is illustrated in Fig. 4A. After adjusting for confounders, each 0.05 increase in ΔFFR_CT_ was independently associated with a greater incidence of early revascularization. Although the predictive value of ΔFFR_CT_ was demonstrated across various subgroups, there was heterogeneity: ΔFFR_CT_ was more predictive for early revascularization in patients with CAD-RADS ≤3, FFR_CT_ 0.71–0.80, or focal and tubular lesions as compared to those with CAD-RADS 4, FFR_CT_ <0.7, or diffuse disease, respectively (Supplemental Figure 3).

### 3.4. Incremental value of ΔFFR_CT_

Receiver operating characteristic curves and the AUC of 3 logistic models for early revascularization are given in Fig. 4B. *Model 2* showed higher AUC compared to *model 1* (0.82 [0.81–0.83] vs. 0.85 [0.84–0.86], p < 0.001). *Model 3* showed a higher AUC compared to *model 2* (0.85 [0.84–0.86] vs. 0.87 [0.86–0.88]), *p* < 0.001), indicating that ΔFFR_CT_ had incremental value to *model 2* for predicting early revascularization. The incremental value of ΔFFR_CT_ was observed across CAD-RADS severities (Supplemental Figure 4A). Heterogenicity of the incremental value was observed according to lesion-specific FFR_CT_. AUC improvement was observed in patients with gray-zone lesion-specific FFR_CT_ of 0.71–0.80, with no difference in those with FFR_CT_ ≤0.70 or FFR_CT_ >0.80 (Supplemental Figure 4B).

### 3.5. ΔFFR_CT_ impact on catheter laboratory utilization

**Fig. 5.**
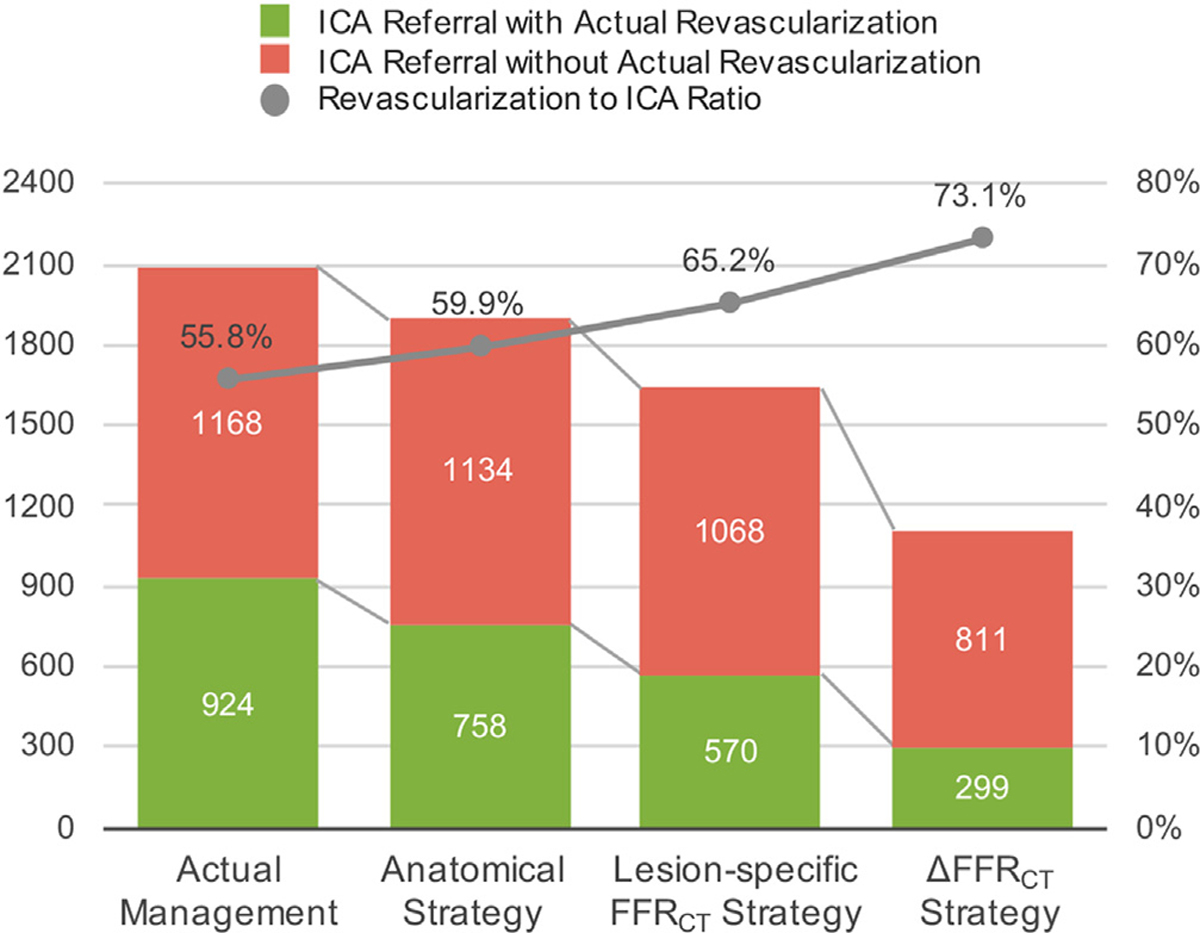
Efficiency of catheter laboratory based on a referral strategy of the 2092 patients who underwent ICA in the ADVANCE registry. Modeling was performed to simulate the efficiency of catheter laboratory utilization according to three ICA referral strategies: *anatomical* strategy based solely on anatomical findings (CAD-RADS ≥3), *lesion-specific FFR*_*CT*_ strategy that also included lesion-specific FFR_CT_ ≤0.80 and a *ΔFFR*_*CT*_ strategy that incorporated ΔFFR_CT_ >0.13 in addition to CAD-RADS ≥3 and lesion-specific FFR_CT_ ≤0.80. Shown are the number of ICA referrals with (green) or without actual revascularization (red) and the ratio of revascularization to ICA according to ICA referral strategy.

A ΔFFR_CT_ of 0.13 was the optimal cut-off for predicting revascularization (Supplemental Figure 5), and we applied this cut-off value to the ICA referral simulation. Actual ICA results and simulated number of ICA and the ratio of subsequent revascularization for each strategy are given in Fig. 5. Although the number of ICA was decreased and ratio of revascularization was increased as compared to actual results, the *anatomical* strategy demonstrated the highest referral for ICA and lowest revascularization ratio among 3 strategies. The *Lesion-specific FFR*_*CT*_ strategy demonstrated a lower number of ICA and higher revascularization ratio as compared to the *anatomical* strategy. The *ΔFFR*_*CT*_ demonstrated the lowest referrals for ICA and the highest revascularization ratio; potentially reducing ICA by 32.2% (1638–1110, *p* < 0.001), and improving the revascularization to ICA ratio from 65.2% [1068/1638] to 73.1% [811/1110] as compared to the lesion-specific FFRCT strategy (Fig. 5). Applying a *ΔFFR*_*CT*_ strategy, the largest improvement in revascularization to ICA ratio was observed in patients with lesion-specific FFR_CT_ between 0.71 and 0.80 (from 43.7% [275/629] to 60.3% [143/237]) as compared to a small improvement in those with an FFR_CT_ of ≤0.70 (from 72.6% [823/1134] to 76.5% [668/873]) (Supplemental Table 2).

## 4. Discussion

This analysis of the ADVANCE registry investigated the utility of ΔFFR_CT_ at predicting early revascularization and discriminating patients with higher revascularization to ICA ratio; both of which may improve efficiency of care of patients with CAD. The main findings of this investigation are as follows: 1) ΔFFR_CT_ values represent a continuum with larger values independently associated with early revascularization, 2) ΔFFR_CT_ demonstrated incremental value at predicting early revascularization compared to a standard strategy of CCTA with lesion-specific FFR_CT_, with the greatest benefit in patients with gray-zone FFR_CT_ values between 0.71 and 0.80, and 3) incorporating ΔFFR_CT_ in addition to standard CCTA and lesion-specific FFR_CT_ diagnostic strategy may reduce the number of ICA and improve the ratio of subsequent revascularization.

While there is increasing evidence supporting the use of FFR_CT_ to improve the efficiency of catheter laboratory utilization,^19–21^ the results of the ADVANCE registry highlight some of the real-world clinical challenges of interpreting FFR_CT_ and guiding downstream decision making. In the ADVANCE registry, 72.3% of patients undergoing ICA with lesion-specific FFR_CT_ of ≤0.80 underwent revascularization.^8^ However, several patients were recommended for medications alone even with positive lesion-specific FFR_CT_ results (<0.80), and some underwent ICA even with negative lesion-specific FFR_CT_ results (0.80), highlighting that there is a space for interpreting the FFR_CT_ results beyond the lesion-specific FFR_CT_. In particular, among patients with lesion-specific FFR_CT_ between 0.71 and 0.80 and who underwent ICA, 56.3% did not subsequently undergo early revascularization. The results of this sub-analysis highlight that ΔFFR_CT_ may improve physician decision-making in identifying patients who require revascularization, particularly those with gray-zone lesion-specific FFR_CT_ values between 0.71 and 0.80. The results of the ISCHEMIA (International Study of Comparative Health Effectiveness with Medical and Invasive Approaches) trial demonstrated that a routine invasive approach does not provide prognostic benefit compared to medical therapy alone.^22^ Accordingly, there is a renewed imperative to consider the risk and benefits of the different treatment options and improve the identification of lesoins that would benefit from revascularization. Given the concerns that a first-line CCTA strategy may result in over referral for ICA,^23^ the ability of ΔFFR_CT_ to identify lesions that require revascularization and potentially improve resource utilization is highly relevant and warrants further investigation with prospective studies.

Our findings also highlight the potential value of non-invasively characterizing the physiological pattern of CAD. Standard lesion-specific FFR_CT_ is affected by coronary atherosclerosis upstream of a measurement point, and the presence of coronary plaque causes FFR_CT_ to decrease even without the obstructive disease.^24^ In contrast to lesion-specific FFR_CT_, ΔFFR_CT_ represents a more stenosis-specific physiological severity and is not affected by coronary plaque beyond the stenosis. Our results suggest that adding ΔFFR_CT_ to lesion-specific FFR_CT_ may inform on disease severity and physiological phenotype. Recent invasive studies have provided similar results with an invasive FFR pullback able to characterize several physiological patterns of CAD.^11,25,26^ A high ΔFFR_CT_ provides an opportunity to identify subjects with a “focal phenotype” of physiology as described by Collet et al.^11^ Despite the clinical benefit observed with FFR-guided PCI,^27^ one-third of patients experience suboptimal post-PCI results, associated with major adverse cardiac events.^28,29^ Therefore, there is an increasing emphasis on achieving a physiologically optimal result post-PCI. In cases with a large focal pressure gradient, PCI is more likely to achieve an ideal functional result and symptomatic benefit for the patient^26^; on the contrary, revascularization in vessels with diffuse pressure loss is associated with limited FFR or symptomatic improvement and even potential harm.^30^ The current approach for reading FFR_CT_ involves interpreting an FFR_CT_ value at one point on the coronary tree, typically 20–30 mm distal to a stenosis (i.e. lesion-specific FFR_CT_).^15^ Although this provides insight into total pressure loss upstream of the coronary artery measurement point, this method is limited in its capacity to characterize the lesion specific physiological phenotype requiring revascularization.^31^ Our results highlight that ΔFFR_CT_ can provide clinically relevant insight into the physiological pattern of disease requiring revascularization. With the emerging use of CCTA and FFR_CT_ to guide PCI,^32^ ΔFFR_CT_ may provide further non-invasive guidance for optimizing revascularization strategies and outcomes.

This study has several limitations. First, the findings related to ΔFFR_CT_ are observational in nature with inherent physician bias for both ICA referral and decisions on revascularization. Second, the endpoints in this study were driven by revascularization. The optimal cut-off value for ΔFFR_CT_ was not powered to evaluate cardiac death and myocardial infarction. However, this does not undermine the opportunity for ΔFFR_CT_ to improve the efficiency of catheter laboratory utilization and adds to recent data from the EMERALD (Exploring the Mechanism of Plaque Rupture in Acute Coronary Syndrome Using Coronary CT Angiography and Computational Fluid Dynamics), demonstrating the prognostic utility of ΔFFR_CT_ at identifying lesions potentially at risk of future myocardial infarction.^10^ Third, revascularization was not guided by invasive FFR. However, the cut-off value derived from the early revascularization as a clinical endpoint was similar to one which compared invasive FFR ≤0.80 or not in previous study,^16^ which may support that the decision for early revascularization was based on coronary physiology in the ADVANCE registry. Finally, the ICA referral simulation did not take into account symptoms and risk factors. The decision to revascularize is multi-factorial, and stenosis and FFR_CT_ are just one part of many factors taken into consideration in the decision making process. Also, the estimates were theoretical, not observed ones. Therefore, further study is warranted. We applied the simulation to patients who underwent ICA to affect clinical factors other than stenosis and FFR_CT_ severity. However, these confounding might not be fully adjusted.

## 5. Conclusions

In this analysis of the ADVANCE registry, ΔFFR_CT_ improves the identification of patients who required early revascularization compared to a standard diagnostic strategy with CCTA and lesion-specific FFR_CT_, with the greatest incremental benefit in patients with gray-zone FFR_CT_ values between 0.71 and 0.80. Applying a criterion of ΔFFR_CT_ to ICA referral, efficiency of resource utilization may be improved. Prospective validation of these findings will be important to translate these findings into broader practice. Appendix A. Supplementary data.

## Supplementary Material

supplemental material

## Figures and Tables

**Table 1:** Patient characteristics.

Variables	Total (n = 4730)	Medication (n = 3562)	Revascularization (n = 1168)	*P*-value

Demographics				
Age, yr	66 ± 10	66 ± 10	66 ± 10	0.678
Female sex, n (%)	1602 (34%)	1285 (36%)	317 (27%)	<0.001
Body mass index, kg/m^2^	26 ± 5	26 ± 5	26 ± 4	0.446
Previous coronary stenting, n (%)	159 (4%)	126 (4%)	33 (3%)	0.389
Angina status, n (%)				<0.001
Typical	1024 (22%)	586 (17%)	438 (38%)	
Atypical	1724 (36%)	1381 (39%)	343 (29%)	
Dyspnea	472 (10%)	375 (11%)	97 (8%)	
Non-cardia Pain	296 (6%)	245 (7%)	51 (4%)	
None	1162 (25%)	928 (26%)	234 (20%)	
Risk factors				
Hypertension, n (%)	2831 (60%)	2091 (59%)	740 (63%)	0.017
Diabetes mellites, n (%)	1034 (22%)	719 (20%)	315 (27%)	<0.001
Hyperlipidemia, n (%)	2749 (58%)	1999 (56%)	750 (64%)	<0.001
Current smoker, n (%)	797 (17%)	560 (16%)	237 (20%)	<0.001

Note. — data are presented as mean ± standard deviation or percentages with raw data in parenthesis. Contentious and categorical variables were compared among groups using the unpaired *t*-test and Fisher’s exact test.

**Table 2:** Coronary artery disease extent.

	Total (n = 4730)	Medications (n = 3562)	Revascularization (n = 1168)	P-value

Anatomical severity				
CAD-RADS, n (%)				<0.001
≤2	1261 (27%)	1227 (34%)	34 (3%)	
3	1773 (38%)	1497 (42%)	276 (24%)	
≥4	1696 (36%)	838 (24%)	858 (73%)	
3-vessel >70% disease	136 (3%)	44 (1%)	92 (8%)	<0.001
Left main >50% disease	163 (3%)	85 (2%)	78 (7%)	<0.001
FFR_CT_ findings				
Minimum lesion-specific FFR_CT_^a^	0.74 ± 0.12	0.77 ± 0.10	0.63 ± 0.11	<0.001
Minimum lesion-specific FFR_CT_, n (%)				
>0.80	1588 (34%)	1518 (43%)	70 (6%)	<0.001
0.71–0.80	1615 (34%)	1340 (38%)	275 (24%)	
≤0.70	1527 (32%)	704 (19%)	823 (70%)	
ΔFFR_CT_^a^	0.13 ± 0.12	0.10 ± 0.09	0.24 ± 0.15	<0.001
Lesion location				<0.001
Left main	798 (17%)	669 (19%)	129 (11%)	
Proximal	1618 (34%)	1166 (32%)	452 (39%)	
Mid	1430 (30%)	1033 (29%)	397 (34%)	
Distal	587 (12%)	460 (13%)	127 (11%)	
Branch	297 (6%)	234 (7%)	63 (5%)	
Stenosis type				<0.001
Focal	4260 (90%)	3271 (92%)	989 (85%)	
Diffuse	470 (10%)	291 (8%)	179 (15%)	

Note. — data are percentages, with raw data in parenthesis, otherwise noted. Contentious and categorical variables were compared among groups using the unpaired *t*-test and Fisher’s exact test. CAD-RADS = coronary artery disease reporting ad data system.

a Data are mean ± standard deviation.

**Table 3:** Multivariable logistic regression analysis for revascularization at 90-day followup.

Predictors	OR (95% CIs)	*P*-value

Patient characteristics		
Age ≥65 yr.	0.83 (0.70–1.00)	0.044
Female sex	0.79 (0.65–0.95)	0.015
Hyperlipidemia	1.40 (1.17–1.68)	<0.001
Diabetes	0.98 (0.80–1.19)	0.811
Hypertension	1.00 (0.83–1.20)	0.999
Current smoker	1.07 (0.86–1.34)	0.526
Angina status (vs. asymptomatic)		
Typical	2.32 (1.83–2.94)	<0.001
Atypical	1.33 (1.06–1.67)	0.014
Non-cardiac	1.19 (0.78–1.80)	0.417
Dyspnea	1.36 (0.98–1.89)	0.067
Imaging findings		
CAD-RADS (ref. ≤2)		
3	4.28 (2.89–6.341)	<0.001
≥4	14.79 (10.04–21.79)	<0.001
FFR_CT_ ≤0.80 (vs. distal)	2.40 (1.49–3.85)	<0.001
ΔFFR_CT_ (per 0.05 increase)	1.47 (1.27–1.70)	<0.001
Lesion location (vs. distal)		
LM	2.09 (1.46–2.99)	<0.001
Proximal	1.81 (1.35–2.42)	<0.001
Mid	1.63 (1.21–2.18)	0.003
Branch	1.48 (0.96–2.27)	0.075
Stenosis type (vs. diffuse)		
Focal	0.82 (0.63–1.07)	0.1433

Note. — *OR* = odds ratio; *CAD-RADS* = Coronary Artery Disease - Reporting and Data System.

## Abbreviations

ADVANCE: assessing diagnostic value of non-invasive FFR_CT_ in coronary care
AUC: area under the receiver operating characteristic curve
CABG: coronary artery bypass grafting
CAD: coronary artery disease
CAD-RADS: Coronary Artery Disease - Reporting and Data System
CCTA: coronary computed tomography angiographZ
FFR: fractional flow reserve
FFR_CT_: fractional flow reserve derived from computed tomography
ICA: Invasive coronary angiography
PCI: percutaneous coronary intervention
