# Supplementary material for: Trans-lesional fractional flow reserve gradient as derived from coronary CT improves patient management: ADVANCE registry☆
Source: J Cardiovasc Comput Tomogr. Author manuscript; Available in PMC 2022 Dec 4. (PMC9719736; doi:10.1016/j.jcct.2021.08.003)
Supplement: supplemental material [file NIHMS1849161-supplement-supplemental_material.docx]

**SUPPLEMENTAL MATERIALS**

Trans-lesional Fractional Flow Reserve Gradient as Derived from Coronary CT Improves Patient Management: ADVANCE registry

Hidenobu Takagi MD PhD^a,b,c^; Jonathon A, Leipsic MD^a^; Noah McNamara^a^, Isabella Martin^a^, Timothy A. Fairbairn MB ChB, PhD^d^, Takashi Akasaka MD^e^; Bjarne L. Nørgaard MD PhD^f^; Daniel S. Berman MD^g^; Kavitha Chinnaiyan MD^h^; Lynne M. Hurwitz-Koweek MD^i^; Gianluca Pontone MD PhD^j^; Tomohiro Kawasaki MD^k^; Niels Peter Rønnow Sand MD PhD^l^; Jesper M. Jensen MD PhD^f^; Tetsuya Amano MD^m^; Michael Poon MD^n^; Kristian A. Øvrehus MD^o^; Jeroen Sonck MD^p,q^; Mark G. Rabbat MD^r^; Sarah Mullen MBT^s^; Bernard De Bruyne MD PhD^p,t^; Campbell Rogers MD^s^; Hitoshi Matsuo MD PhD^u^; Jeroen J. Bax MD PhD^v^; Pamela S. Douglas, MD^i^; Manesh R. Patel MD^i^; Koen Nieman MD PhD^w^; Abdul Rahman Ihdayhid MBBS PhD^a,x^

^a^Department of Radiology, St. Paul’s Hospital and University of British Columbia, Vancouver, British Columbia, Canada

^b^Department of Radiology, Iwate Medical University Hospital, Iwate, Japan

^c^Department of Diagnostic Radiology, Tohoku University Hospital, Miyagi, Japan

^d^Department of Cardiology, Liverpool Heart and Chest Hospital, University of Liverpool, Liverpool, United Kingdom

^e^Department of Cardiovascular Medicine, Wakayama Medical University, Wakayama, Japan

^f^Department of Cardiology, Aarhus University Hospital, Aarhus, Denmark

^g^Division of Nuclear Imaging, Department of Imaging, Cedars-Sinai Heart Institute, Los Angeles, CA, USA

^h^Division of Cardiology, Beaumont Academic Heart and Vascular Group, Royal Oak, MI, USA

^i^Division of Cardiology, Department of Medicine, Duke University Medical Center, Duke Clinical Research Institute, Duke University School of Medicine, Durham, NC, USA

^j^Centro Cardiologico Monzino, IRCCS, Milan, Italy

^k^Cardiovascular Center, Shin Koga Hospital, Fukuoka, Japan

^l^Cardiac Research Unit, Institute of Regional Health Research, University Hospital of Southern DK, Esbjerg and University of Southern DK, Denmark

^m^Department of Cardiology, Aichi Medical University, Aichi, Japan

^n^Department of Noninvasive Cardiac Imaging, Northwell Health, New York, NY, USA

^o^Department of Cardiology, Odense University Hospital, Denmark

^p^Cardiovascular Center Aalst, OLV Clinic, Aalst, Belgium

^q^Department of Advanced Biomedical Sciences, University of Naples Federico II, Naples, Italy

^r^Division of Cardiology, Loyola University Chicago, Chicago, IL, USA

^s^HeartFlow Inc., Redwood City, CA, USA

^t^Department of Cardiology, University Hospital of Lausanne, Lausanne, CH

^u^Department of Cardiovascular Medicine, Gifu Heart Center, Gifu, Japan

^v^Department of Cardiology, Leiden University Medical Center, Leiden, the Netherlands

^w^Departments of Cardiovascular Medicine and Radiology, Stanford University, Stanford, California

^x^Department of Cardiology, Fiona Stanley Hospital, Harry Perkins Institute of Medical Research, University of Western Australia, Perth, Australia.

Table of Contents

[Supplemental Documents 3](#_Toc76223903)

[Inter-observer reproducibility of ΔFFR_CT_ 3](#_Toc76223904)

[Supplemental Tables 4](#_Toc76223905)

[**Supplemental Table 1**. Definition of CAD-RADS grading 4](#_Toc76223906)

[**Supplemental Table 2**. The simulated ratio of revascularization to ICA 5](#_Toc76223907)

[Supplemental Figures 6](#_Toc76223908)

[**Supplemental Figure 1**. Patient selection 6](#_Toc76223909)

[**Supplemental Figure 2**. Relationship of ΔFFR_CT_ with CAD-RADS (**A**) and FFR_CT_ (**B**) 7](#_Toc76223910)

[**Supplemental Figure 3**. Heterogeneity of predictive value of ΔFFR_CT_ for early revascularization 8](#_Toc76223911)

[**Supplemental Figure 4**. Receiver operating characteristic curve and area under the curve according to CAD-RADS (**A**) and lesion-specific FFR_CT_ severity (**B**) 9](#_Toc76223912)

[**Supplemental Figure 5**. 10](#_Toc76223913)

# **Supplemental Documents**

## Inter-observer reproducibility of ΔFFR_CT_

The inter-observer reproducibility of the ΔFFR_CT_ was assessed in 40 randomly selected patients using an intraclass correlation coefficient (ICC) and mean difference with 95% confidence interval. The ICC was excellent on per-patient and vessel level, which was comparable to one in which the reference points were determined on coronary CT angiography images (1). No significant inter-observer difference was observed for per-patient, LAD-, LCX-, and LM-level. Although statistically significant, the mean difference for RCA was small.

|  | ICC (95% CI) | Mean difference (95% CI) | *P* value^*^ |
| --- | --- | --- | --- |
| Per-patient | 0.97 (0.94–0.98) | 0.01 (-0.01–0.02) | 0.303 |
| RCA | 0.95 (0.90–0.97) | 0.01 (0.00–0.02) | 0.033 |
| LAD | 0.94 (0.89–0.97) | 0.01 (0.00–0.02) | 0.111 |
| LCX | 0.98 (0.97–0.99) | 0.00 (-0.01–0.01) | 0.868 |
| LM | 0.84 (0.72–0.91) | 0.00 (-0.01–0.00) | 0.480 |

Note. — RCA -= right coronary artery; LAD = left anterior descending artery; LCX = left circumflex artery; and LM = left main

* mean difference was compared by paired t test.

**Reference**

1. Takagi H., Ishikawa Y., Orii M., et al. Optimized interpretation of fractional flow reserve derived from computed tomography: Comparison of three interpretation methods. J Cardiovasc Comput Tomogr 2019;2(13):134–41. Doi: 10.1016/j.jcct.2018.10.027.

# **Supplemental Tables**

## **Supplemental Table 1**. Definition of CAD-RADS grading

| Grading | Degree of maximal coronary stenosis |
| --- | --- |
| CAD-RADS 0 | 0% (No plaque or stenosis) |
| CAD-RADS 1 | 1–24% stenosis |
| CAD-RADS 2 | 25–49% stenosis |
| CAD-RADS 3 | 50–69% stenosis |
| CAD-RADS 4A | 70–99% stenosis |
| CAD-RADS 4B | Left main >50% or 3- vessel obstructive (≥70%) disease |
| CAD-RADS 5 | 100% (total occlusion) |

## **Supplemental Table 2**. The simulated ratio of revascularization to ICA

|  | Actual | Anatomical  strategy^*^ | Lesion-specific  FFR_CT_ strategy^*^ | ΔFFR_CT_  strategy^*^ |
| --- | --- | --- | --- | --- |
| Total | 55.8% (1168/2092) | 59.9% (1134/1892) | 65.2% (1068/1638) | 73.1% (811/1110) |
| CAD-RADS |  |  |  |  |
| ≤2 | 17.0% (34/200) | NA | NA | NA |
| 3 | 40.5% (276/682) | 40.5% (276/682) | 46.0% (248/539) | 53.4% (167/313) |
| ≥4 | 70.9% (858/1210) | 70.9% (858/1210) | 74.6% (820/1099) | 80.8% (644/797) |
| Lesion-specific FFR_CT_ |  |  |  |  |
| >0.80 | 21.3% (70/329) | 26.0% (66/254) | NA | NA |
| 0.71–0.80 | 43.7% (275/629) | 47.2% (260/551) | 47.2% (260/551) | 60.3% (143/237) |
| ≤0.70 | 72.6% (823/1134) | 74.3% (808/1087) | 74.3% (808/1087) | 76.5% (668/873) |
| Angina status |  |  |  |  |
| Typical | 66.4% (438/660) | 69.2% (429/620) | 74.6% (416/558) | 81.2% (328/404) |
| Non-typical | 51.0% (730/1432) | 55.4% (705/1272) | 60.4% (652/1080) | 68.4% (483/706) |
| Lesion location |  |  |  |  |
| Left main | 50.0% (129/258) | 56.7% (127/224) | 63.8% (113/177) | 75.4% (52/69) |
| Proximal | 57.0% (452/793) | 61.1% (442/723) | 66.0% (422/639) | 73.9% (320/433) |
| Mid | 58.1% (397/683) | 61.1% (382/625) | 66.6% (357/536) | 73.8% (293/397) |
| Distal or branch | 53.1% (190/358) | 57.2% (183/320) | 61.5% (176/286) | 69.2% (146/211) |

Note. – values are simulated ratio of revascularization to ICA, with a raw number of actual revascularization and simulated ICA referral in parentheses. ICA = invasive coronary angiography; CAD-RADS = coronary artery disease – reporting and data system; FFR_CT_ = fractional flow reserve derived from coronary CT angiography; NA = not applicable.

****Anatomical*** strategy, patients with CAD-RADS ≥3 undergo ICA; ***lesion-specific FFR_CT_*** strategy, patients with CAD-RADS ≥3 and FFR_CT_ ≤0.80 undergo ICA; and ***ΔFFR_CT_***

strategy, patients with CAD-RADS ≥3 and FFR_CT_ ≤0.80 and ΔFFR_CT_ >0.13 undergo ICA.

# **Supplemental Figures**

## **Supplemental Figure 1**. Patient selection


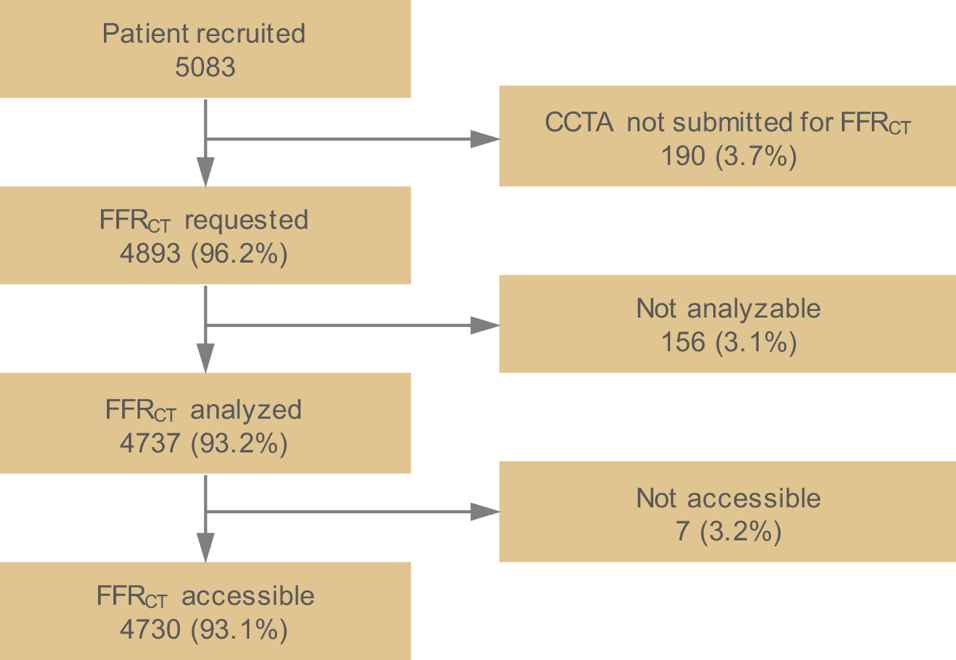


Flow chart represents patient selectin.

CCTA = coronary computed tomography angiography; FFR_CT_ = fractional flow reserve derived from CT

## **Supplemental Figure 2**. Relationship of ΔFFR_CT_ with CAD-RADS (**A**) and FFR_CT_ (**B**)


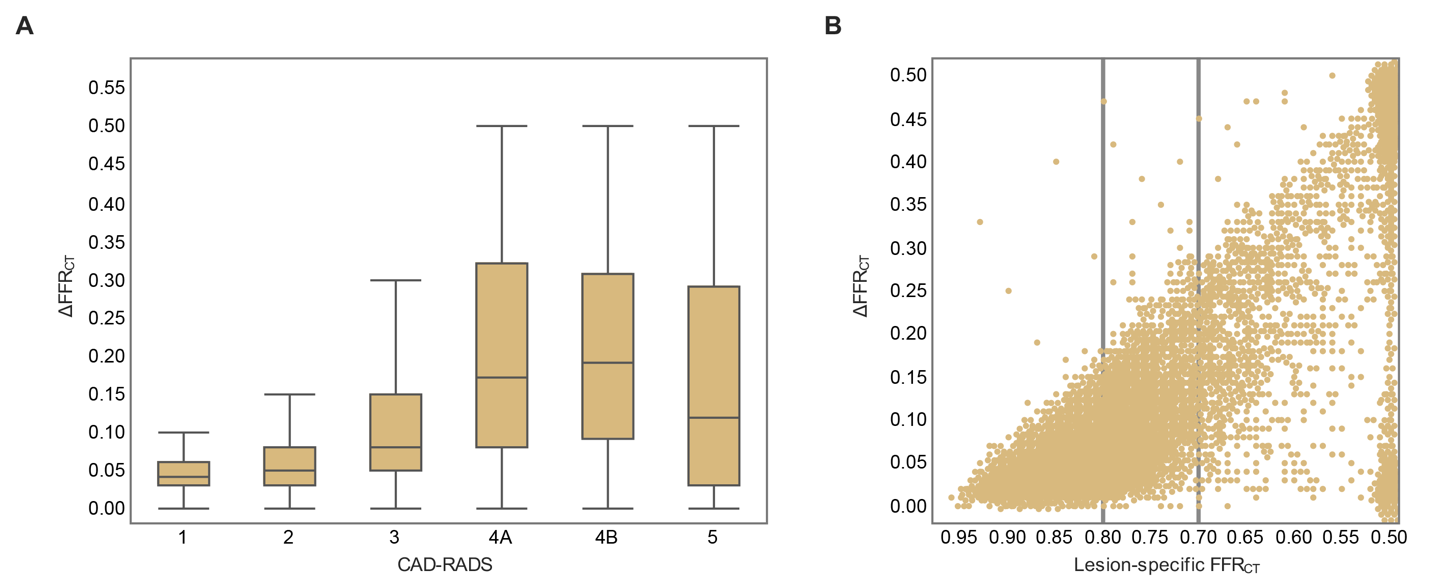


ΔFFR_CT_ increased with increasing CAD-RADS: CAD-RADS 1, 0.05 ± 0.05; 2, 0.07 ± 0.07; 3, 0.12 ± 0.10; 4A, 0.21 ± 0.15; 4B, 0.21 ± 0.14; 5, 0.17 ± 0.16 (**A**). There was significant inverse correlation between lesion-specific FFR_CT_ and ΔFFR_CT_ (Spearman’s rho, -0.68 [95% CI, -0.70–-0.66], *p* <0.001) (**B**).

## **Supplemental Figure 3**. Heterogeneity of predictive value of ΔFFR_CT_ for early revascularization

*P* value is for interaction between the interest subgroups and ΔFFR_CT_ and calculated in a multivariable logistic regression analysis adjusted for risk factors, stenosis type and location, and CAD-RADS. *P* values are reported without adjustment for multiplicity of testing. Adjusted odds ratio (OR) (**square**) with 95% confidence interval (CI) (**line**) are reported for per 0.05-unit ΔFFR_CT_ increase.

## **Supplemental Figure 4**. Receiver operating characteristic curve and area under the curve according to CAD-RADS (**A**) and lesion-specific FFR_CT_ severity (**B**)

The incremental discrimination for predicting early revascularization was compared among three models (***model 1***: risk factors + stenosis type and location + CAD-RADS; ***model 2***: ***model 1*** + FFR_CT_; and ***model 3***: ***model 2*** + ΔFFR_CT_) in each CAD-RADS and lesion-specific FFR_CT_ category. Shown numbers are area under the curve (AUC) with 95% confidence intervals in parenthesis. AUCs were compared among between models by DeLong’s test.

* means significant difference compared with ***model 1***

† means significant difference compared with ***model 2***

## **Supplemental Figure 5**.
